# Effects of thrombin, PAR-1 activating peptide and a PAR-1 antagonist on umbilical artery resistance in vitro

**DOI:** 10.1186/1477-7827-3-8

**Published:** 2005-02-24

**Authors:** Aonghus J O'Loughlin, Crochan J O'Sullivan, Nandini Ravikumar, Anne M Friel, John T Elliott, John J Morrison

**Affiliations:** 1 Dept. of Obstetrics & Gynaecology, National University of Ireland Galway, Clinical Science Institute, University College Hospital Galway, Newcastle Road, Galway, Ireland; 2 Biotechnology Division/Biomaterials Group, NIST, 100 Bureau Dr. Bldg 227 Rm A249, Gaithersburg, MD 20899-8313, USA

## Abstract

**Background:**

The non-thrombotic effects of thrombin in cardiovascular tissues, as mediated via the protease activated receptors (PARs), and particularly PAR-1, have been the focus of much recent research. The aims of this study were to evaluate the effects of thrombin, a specific PAR-1 activating peptide (PAR1-AP), and a PAR-1 antagonist on human umbilical artery tone in vitro.

**Methods:**

Human umbilical artery samples were obtained from 17 women at term. Arterial rings were suspended under physiologic conditions for isometric recording. The in vitro effects of thrombin (0.5 units/mL to 3 units/mL), PAR1-AP TFLLR-NH2 [10(-9) to 10(-6) M], and PAR-1 antagonist (N-trans cinnamoyl- p-fluoroPhe-p-guanidinoPhe-Leu-Arg-Orn-NH2) [10(-9) M to 10(-5) M] on umbilical artery tone were measured.

**Results:**

Both thrombin and TFLLR-NH2 exerted a potent cumulative vasodilatory effect on human umbilical artery resistance (P < 0.001). The mean net maximal inhibition (MMI) for thrombin was 53.05% (n = 6; SEM = 1.43) at tissue bath concentration of 3 units/mL. The MMI with TFLLR-NH2 was 61.50 % (n = 6; SEM = 1.43) at bath concentration of 10(-6) M. In comparison to vehicle control, the PAR-1 antagonist did not show a significant relaxant or contractile effect (P > 0.05).

**Conclusion:**

These findings highlight a potential role for thrombin and PAR-1 receptors in vascular regulation of feto-placental blood flow in normal pregnancy, and in association with the vascular lesions associated with IUGR and pre-eclampsia.

## Background

In disorders resulting in poor fetal growth, and in pre-eclampsia, thrombotic lesions are frequently observed in the maternal and fetal vascular components of the placenta, [1-3] and hence have been implicated in the pathophysiology of these conditions. In addition, it has been reported that in vivo generation of thrombin, in maternal plasma, is higher in patients with small for gestation age fetuses and with pre-eclampsia, than in normal pregnancy [4]. It is well established, for many years, that thrombin plays a role in blood coagulation, but its effects in many other cell and tissue types (smooth muscle cells, endothelial cells, lymphocytes) [5,6] have been the subject of more recent attention. It is now apparent that thrombin can regulate target cells by cleaving and activating a family of G-protein-coupled protease-activated receptors (PARs)[5-7]. This proteolytic cleavage of PARs is mediated by a family of enzymes that require serine within the active site i.e. serine proteases [5]. There are 4 major PAR subtypes (PAR1-4) with diverse reported functions in various tissues [6]. PAR activation has been closely linked to inflammation [6,8], contraction of vascular [5] and non-vascular [9,10] smooth muscle, and platelet activation [6]. Although PAR-1, PAR-3 and PAR-4 [11] are all known to be thrombin receptors, the mechanism of activation by thrombin at these different PARs varies [12,13].

There is growing evidence, from vascular tissue studies in several animal models, that non-thrombotic thrombin-mediated signalling events are central to the response to the disease process typical of vascular lesion formation in atherosclerosis [14]. The direct effects of thrombin on vascular cells, via the PAR receptors, and particularly PAR-1, have been the main focus of investigation for this hypothesis. PARs 1, 3 and 4 are activated by thrombin [11], but PAR-1 is activated at low thrombin concentrations and most of what is known about thrombin signalling downstream of the receptors, has been derived from studies of PAR-1 [14]. While other PAR subtypes are present in human arterial vessels, it appears that PAR-1 is primarily involved in endothelium-dependent relaxation to thrombin and trypsin [15]. To our knowledge, there are no data outlining the potential effects of thrombin, or specific PAR-1 receptor modulation, on the feto-placental circulation, despite the critical role of thrombin in disorders of this vasculature. The aims of this study were to evaluate the direct effects of thrombin, the specific PAR-1 activating peptide (PAR1-AP), TFLLR-NH_2 _(Thr-Phe-Leu-Leu-Arg-NH_2_), and the PAR-1 specific antagonist (N-trans cinnamoyl -p-fluoroPhe-p-quanidinoPhe-Leu-Arg-Orn-NH_2_) on human umbilical artery tone in vitro.

## Methods

The study was carried out in the Department of Obstetrics and Gynaecology, University College Hospital Galway, Ireland between May 2002 and April 2003. Sections of human umbilical cord approximately 10 cm in length were excised from the proximal segment of the cord (i.e. closest to the placental attachment) immediately after elective cesarean delivery. Samples were obtained from 17 women after elective cesarean section at term and from one patient after normal vaginal delivery. All pregnancies were uncomplicated and there was no evidence of hypertensive disease or intrauterine fetal growth restriction. The mean maternal age was 32.47 years (range 25–40 years). The median period of gestation was 38 weeks (range 37–42 weeks). The reasons for cesarean section included previous cesarean section (n = 9), breech presentation (n = 6), previous myomectomy (n = 1) and unstable fetal lie (n = 1). At the time of recruitment 4 women were nulliparous and 13 women were parous.

Samples were immediately placed in cold buffered Krebs Henseleit physiological salt solution (pH 7.4) of the following composition: potassium chloride 4.7 mmol/L, sodium chloride 118 mmol/L, magnesium sulphate 1.2 mmol/L, calcium chloride 1.2 mmol/L, potassium phosphate 1.2 mmol/L, sodium bicarbonate 25 mmol/L and glucose 11 mmol/L. Maternal written informed consent was obtained prior to tissue collection, and the tissue collection procedure was approved by the Research Ethics Committee at University College Hospital Galway.

Umbilical arteries were carefully dissected free of Wharton's Jelly and cut in rings 4–5 mm in axial length. Rings were suspended individually on stainless steel hooks inserted into their lumens and mounted under 2 g (30 mN) of isometric tension, in glass-jacketed tissue baths, as previously described [16,17]. Each bath-contained 10 mL of oxygenated (95% O_2 _/ 5% CO_2_) Krebs Henseleit physiological salt solution (PSS) at 37°C and pH 7.4. Rings were allowed to equilibrate for 90 minutes with regular washouts of PSS. During this interval, spontaneous tone developed. After the equilibration period, the vessel rings were challenged with 60 mM potassium chloride (KCl). Three washouts with PSS were carried out once the maximum response had reached a plateau, and a 20 minute recovery period was allowed in order that baseline be attained again. The KCl challenge was performed three times. After the last KCl challenge, 40 minutes recovery was allowed, and contraction was then stimulated by bath exposure of the vessel rings to 5-hydroxytriptamine (5-HT) (10^-7 ^M). Once maximum contractile response to 5-HT was attained, the rings were allowed to remain at plateau for 20 minutes. Concentration- effect experiments were performed by cumulative additions of thrombin, the PAR1-AP, or the PAR-1 antagonist to the tissue bath. Thrombin was added to the tissue bath at an initial concentration of 0.5 units/mL, and this was increased at 20-minute intervals to 1 unit/mL, 2 units/mL and 3 units/mL respectively. The bath concentrations ranges investigated for PAR1-AP were 1 nanomol/L, 10 nanomol/L, 100 nanomol/L and 1 micromol/L (i.e. 10^-9 ^- 10^-6^M), and for PAR-1 antagonist were 1 nanomol/L, 10 nanomol/L, 100 nanomol/L, 1 micromol/L and 10 micromol/L (i.e. 10^-9^-10^-5^M), all at 20-minute intervals.

The effects of thrombin, PAR1-AP and PAR-1 antagonist were demonstrated by expressing the mean amplitude calculated during the 20-minute period following addition of each drug concentration, as a percentage of the mean amplitude obtained in the 20 minutes prior to any drug addition. This measurement represents percentage contractility or tone, and subtracted from 100%, provides the percentage relaxation value for each bath concentration of vehicle and study compounds. The net relaxant effect of each compound was calculated by subtracting the percentage contractility value calculated for its respective vehicle (for thrombin, PAR1-AP or PAR-1 antagonist), control experiment, at each similar bath concentration. All of the umbilical artery samples used for experimentation for each compound were obtained from different women (i.e. n = 6 for PAR1-AP), for example, was achieved by using umbilical artery samples from 6 different women). The allocation of umbilical artery samples for the different experiments was entirely random.

Fresh Krebs Henseleit physiological salt solution was made and buffered daily. KCl solutions were prepared on the day of experimentation. A stock solution of 5-HT (Sigma-Aldrich, Dublin, Ireland) was made up in de-ionised water and diluted with Krebs solution. Thrombin was purchased from Sigma-Aldrich (Dublin, Ireland) and a stock solution of 1100 U/ml prepared in de-ionised water and stored at -20°C. PAR1-AP was purchased from Tocris Cookson Ltd (Bristol, UK) and a stock solution of 1 millimol/L was prepared using deionized water, with subsequent dilutions in PSS. The PAR-1 antagonist was prepared and assayed by methods previously described [18]. The IC_50 _value for antagonist inhibition of platelet aggregation stimulated with 1 micromol/L SFLLRN agonist was determined to be 0.1 micromol/L. It was prepared as a 10 millimol/L stock solution in dimethysulphoxide (DMSO) and stored in room temperature protected from direct light. Final bath concentrations of DMSO, at the highest concentration of PAR-1 antagonist, did not exceed 1%, for both study and vehicle control strips. Serial dilutions of thrombin, PAR1-AP and the PAR-1 antagonist were made using Krebs Henseleit physiological salt solutions.

Comparisons of measurements of amplitude for each bath concentration of thrombin, PAR1-AP and the PAR-1 antagonist, or respective control vehicle, were made using a one-way ANOVA. Post-hoc comparisons were made using the Tukey HSD test. A P value <0.05 was accepted as statistically significant. The statistical package SPSS version 10 was used for statistical calculations.

## Results

**Figure 1:**
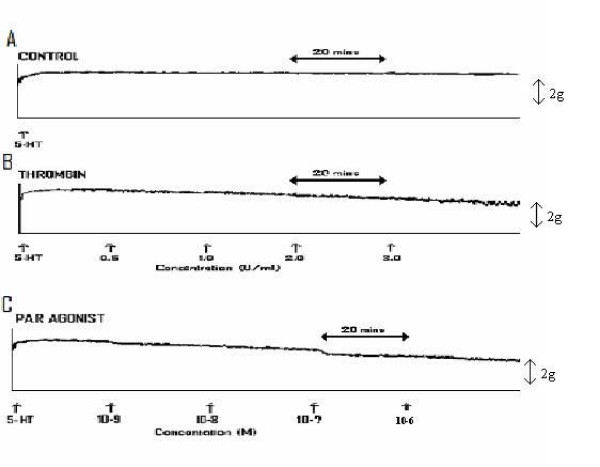
Representative recording of **A) **Serotonin induced contraction of umbilical artery, **B) **Serotonin induced contraction followed by cumulative additions of Thrombin and **C) **Serotonin induced contraction followed by cumulative additions of PAR1-AP.

A recording from control experiments (i.e. without addition of vehicle, thrombin, PAR1-AP or PAR-1 antagonist) demonstrating umbilical artery tone due to bath exposure of the ring to serotonin, for the entire duration of an experiment, is shown in Figure 1A. The mean net spontaneous relaxation of tone observed was 14.39% (SEM = 2.76). Thrombin exerted a potent and cumulative vasodilatory effect on umbilical artery tone in comparison to simultaneous vehicle (PSS) only experiments. A representative recording of the effects of thrombin is shown in Figure 1B. At bath concentrations at or greater than 0.5 units/mL thrombin exerted a significant vasodilatory effect. The net vasodilatory effects of thrombin are provided in Table 1. The mean net inhibition of tone observed, at maximum thrombin concentration investigated (i.e. 3 units/mL) was 53.5% (SEM = 4.62; n = 6; P < 0.001).

**Table 1:** Net inhibitory effect of thrombin, PAR-1 and PAR1-AP on human umbilical artery tone.

**Thrombin (n = 6)**	**(% ± SEM)**	**PAR1-AP (n = 6)**	**(% ± SEM)**	**PAR-1 Antagonist (n = 6)**	**(% ± SEM)**	**(P Value)**
***0.5 U/ml***	#28.90 ± 2.60	***10^-9^M***	*23.91 ± 5.64	***10^-9^M***	2.83 ± 3.05 (NS)	(0.630)
***1 U/ml***	*35.39 ± 3.91	***10^-8^M***	*37.32 ± 2.29	***10^-8^M***	3.80 ± 2.33 (NS)	(0.471)
***2 U/ml***	*44.72 ± 2.31	***10^-7^M***	*52.39 ± 1.28	***10^-7^M***	5.60 ± 3.74 (NS)	(0.228)
***3 U/ml***	*53.51 ± 4.62	***10^-6^M***	*61.50 ± 1.43	***10^-6^M***	7.03 ± 5.74 (NS)	(0.148)
				***10^-5^M***	6.87 ± 4.48 (NS)	(0.815)

Values presented represent the mean inhibitory effects on umbilical artery tone i.e. after adjusting for control / vehicle experiments. The values provided represent % inhibition ± standard error of the mean (SEM). The PAR1-AP used was Threonine-Phenylalanine-Leucine-leucine-Arginine-NH_2_. The PAR-1 antagonist used was Ser- pFPhe-pGPe-Leu-Arg-Orn-NH_2_. The values were compared with amplitude measurements observed prior to drug addition (NS not significant, # P < 0.01, * P < 0.001).

TFLLR-NH_2_, the PAR1-AP, similarly exerted a cumulative vasodilatory effect on umbilical artery tone. A representative recording of the cumulative effects of PAR1-AP are shown in Figure 1C, with the mean difference in amplitude measurements (i.e. in comparison to simultaneous control experiments) provided in Table 1. The mean net inhibition of tone observed, at maximum PAR1-AP concentration investigated (i.e. 10^-6^M / 1 micromol/L) was 61.5% (SEM = 1.43; n= 6; P < 0.001).

**Figure 2:**
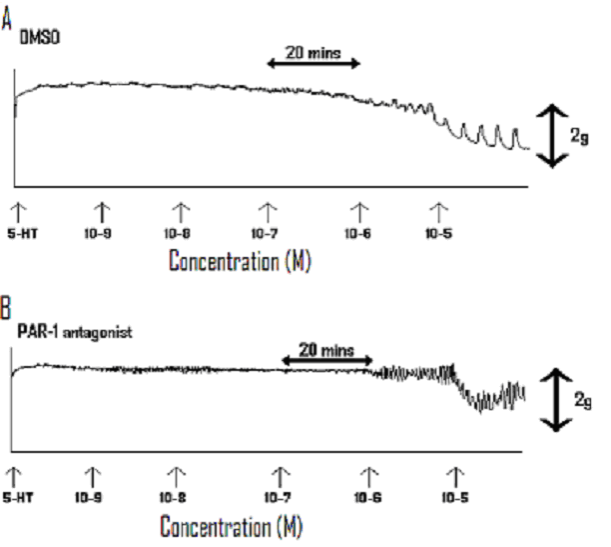
Representative recordings of **A) **umbilical artery tone after serotonin induced contraction with exposure to vehicle only (i.e. DMSO added cumulatively), and **B) **the effects of cumulatively increasing bath exposure of arterial rings to Ser- pFPhe-pGPe-Leu-Arg-Orn-NH_2 _after serotonin induced contraction.

For the PAR-1 antagonist, N-trans cinnamoyl-p-fluoroPhe-p-guanidinoPhe-Leu-Arg-Orn-NH_2_, no relaxation of umbilical artery tone in vitro was observed, in comparison to vehicle only control experiments. Figure 2A demonstrates a representative recording of umbilical artery tone, after exposure to vehicle only (i.e. DMSO added cumulatively). The mean net relaxation of tone observed with vehicle was 70.28% (SEM = 2.98). In Figure 2B, a representative recording of the effects of cumulatively increasing bath exposure of arterial rings to the PAR-1 antagonist is shown. The mean net maximal inhibition exerted was 6.87% (SEM = 1.57; n = 6; P = 0.280). Finally, there was no observed difference in the vasodilatory effects of thrombin, PAR1-AP or the PAR-1 antagonist in relation to parity.

## Discussion

This study demonstrates that thrombin exerts a potent vasodilatory effect on human umbilical artery vasculature in vitro. While it is known that thrombin has an inhibitory effect on vessel tone in other animal and human vascular tissue types [19-21] this is the first report, to our knowledge, of the direct effects of thrombin on human umbilical artery vasculature. Receptors for thrombin, the PAR family of receptors, are present on vascular smooth muscle cells, and on endothelial cells [14,22], with the effects of thrombin mediated mainly via the PAR 1, 3 and 4 receptor subtypes [11]. The potential physiological function of thrombin in mediating vascular tone in the umbilical circulation in normal pregnancy is unknown. Our findings also raise questions in relation to the role of the non-thrombotic effects of thrombin in the feto-placental vasculature in disorders of pregnancy such as pre-eclampsia and intrauterine growth restriction, which are classically associated with thrombotic lesions or a relative excess of thrombin [1-3]. The possibility that the vasodilatory effect of thrombin may serve to counteract the diminished perfusion associated with the pathophysiology of these conditions, at least in the early stages of disease, is one hypothesis. A further theory is that the much enhanced uterine contractility elicited by thrombin and PAR1-AP [13] may be concomitantly associated with a feto-placental vasodilatory effect to maintain good utero-placental blood flow.

We have not elucidated the exact mechanism, or mechanisms, of the thrombin mediated alteration in vascular tone in human umbilical artery. We have however demonstrated that a similar effect is elicited by bath exposure of the arterial rings to the specific PAR1-AP, TFLLR-NH_2_. PAR1-APs are recently designed small synthetic peptide ligands which mimic the effects of proteases by binding directly to the activation site of the PAR-1 receptor, bypassing the need for proteolytic cleavage of the receptor. Soluble peptide ligands, as PAR-APs are, can vary greatly in potency as agonists in comparison with proteases [10]. TFLLR-NH_2 _is reportedly one of the more selective PAR1-APs and is deemed most preferable for purposes of studying the physiologic role of PAR-1 [10]. In view of the fact that PAR-1 function is central to a thrombin-mediated effect in other tissues [11,14,15] and that a PAR-1 mediated effect in human umbilical artery vasculature is similar to that elicited by thrombin, the findings from this study are suggestive of a major role for PAR-1 in mediating the vasodilatory effect of thrombin in umbilical artery vasculature. Other possible mechanisms, and the potential role of PARs 3 and 4, have not been evaluated in this study.

The PAR-1 antagonist, N-trans cinnamoyl-p-fluoroPhe-p-guanidinoPhe-Leu-Arg-Orn-NH_2_, did not alter umbilical artery tone. This peptide is a competitive antagonist i.e., it competes for the agonist binding site and does not appear to have any other activity [23]. The direct effects of PAR-antagonism had not previously been evaluated on umbilical artery preparations in vitro, and hence their inclusion in this study. We have also previously demonstrated that this specific PAR-1 antagonist exerted a relaxant effect on human myometrium in vitro, the mechanism of which is unknown [24]. The results observed here serve to confirm that N-trans cinnamoyl-p-fluoroPhe-p-guanidinoPhe-Leu-Arg-Orn-NH_2 _is inactive towards the PAR-1 receptor. The true role of PAR antagonists, in such experiments, is for the purpose of investigation of the potential effects of an agonist at a PAR subtype. Pre-incubation of the arterial rings with PAR-1 antagonist, for example, should alter the response elicited by PAR1-AP. Such experiments, with selective blockade of the PAR subtypes, in normal and diseased pregnancies, are a subject of further studies.

There are some limitations to this study. All umbilical cord artery samples were obtained at elective cesarean section. This was included in the design of the study in order to maintain uniformity in terms of mode of delivery, and, to avoid using cord samples that may have undergone excessive traction in the third stage of labor at vaginal delivery. It is unknown whether this latter point is valid, or not, in studies using in vitro umbilical preparations. We are currently performing comparative studies from samples obtained at vaginal delivery to further evaluate this matter. Secondly, the effects of thrombin and PAR-1 modulation, on smaller vessels in the feto-placenta circulation (i.e. placental arteries), and in association with disorders of pregnancy, would compliment our knowledge of the importance of this pathway. Finally, there are limitations in extrapolating from in vitro experiments to the in vivo situation, but the experiments conducted here represent a reliable and valid in vitro model for these vascular preparations.

## Conclusion

In conclusion, thrombin exerts a potent vasodilatory effect on umbilical artery preparations in vitro. A similar effect is also observed using a specific PAR1-AP. The potential non-thrombotic role of thrombin, and PAR subtype modulation, in regulation of the feto-placental circulation in normal pregnancy, and in pregnancies complicated by hypertensive disease or intrauterine growth restriction, is highlighted by these findings.

## Authors' contributions

AJO'L performed the experiments and wrote the manuscript. CJO'S and NR performed the experiments. AMF analysed the data and wrote the manuscript. JTE provided the PAR-1 antagonist (N-trans cinnamoyl- p-fluoroPhe-p-guanidinoPhe-Leu-Arg-Orn-NH_2_) and wrote the manuscript. JJM designed, supervised the study and wrote the manuscript. All authors read and approved the final manuscript.

## References

[B1] Rayne SC, Kraus FT (1993). Placental thrombin and other vascular lesions. Classification, morphology, and clinical correlations. Pathol Res Pract.

[B2] Salafia CM, Minior VK, Pezullo JC, Popek EJ, Rosenkrantz TS, Vintzileos AM (1995). Intrauterine growth restriction in infants of less than thirty-two weeks gestation: associated placental pathological features. Am J Obstet Gynecol.

[B3] Mitra SC, Seshan SV, Riachi LE (1998). Placental vessel morphometry in growth retardation and increased resistance of the umbilical artery Doppler flow. J Matern Fetal Med.

[B4] Chaiworapongsa T, Yoshimatsu J, Espinoza J, Kim YM, Berman S, Edwin S, Yoon BH, Romero R (2002). Evidence if in vivo generation of thrombin in patients with small-for-gestational-age fetuses and pre-eclampsia. J Matern Fetal Neonatal Med.

[B5] Macfarlane SR, Seatter MJ, Kanke T, Hunter GD (2001). Plevin Proteinase-activated receptors. Pharmacol Rev.

[B6] Vergnolle N, Wallace JL, Bunnett NW, Hollenberg MD (2001). Protease-activated receptors in inflammation, neuronal signalling and pain. Trends Pharmacol Sci.

[B7] Hollenberg MD (1996). Protease-mediated signalling: new paradigms for cell regulation and drug development. Trends Pharmacol Sci.

[B8] Cocks TM, Moffatt JD (2000). Protease-activated receptors: sentries for inflammation?. Trends Pharmacol Sci.

[B9] Cicala C, Bucci M, De Dominicis G, Harriot P, Sorrentino L, Cirino G (1999). Bronchoconstrictor effect of thrombin and thrombin receptor activating peptide in guinea-pigs in vivo. Br J Pharmacol.

[B10] Vergnolle N (2000). Review article: protease-activated receptors – novel signals for gastrointestinal pathophysiology. Aliment Pharmacol Ther.

[B11] Coughlin SR (1999). How the protease thrombin talks to cells. Proc Natl Acad Sci USA.

[B12] Coughlin SR (2000). Thrombin signalling and protease-activated receptors. Nature.

[B13] O'Sullivan CJ, Allen NM, O'Loughlin AJ, Friel AM, Morrison JJ (2004). Thrombin and PAR-1 Activating peptide: Effects on human uterine contractility in vitro. Am J Obstet Gynecol.

[B14] Patterson C, Stouffer GA, Madamanchi N, Runge MS (2001). New tricks for old dogs: Non-thrombotic effects of thrombin in vessel wall biology. Circ Res.

[B15] Hamilton JR, Moffatt JD, Frauman AG, Cocks TM (2001). Protease-activated receptor (PAR) 1 but not PAR2 or PAR4 mediates endothelium-dependent relaxation to thrombin and trypsin in human pulmonary arteries. J Cardiovasc Pharmacol.

[B16] Dennedy MC, Houlihan DD, McMillan HM, Morrison JJ, β2 and β3-adrenoceptor agonists (2002). Human myometrial selectivity and effects on umbilical artery tone. Am J Obstet Gynecol.

[B17] Potter SM, Dennedy MC, Morrison JJ (2002). Corticosteroids and fetal vasculature: Effects of hydrocortisone, dexamethasone and betamethasone on human umbilical artery. BJOG.

[B18] Elliott JT, Hoekstra WJ, Maryanoff BE, Prestwich GD (1999). Photoactivatable peptides based on BMS-197525: a potent antagonist of the human thrombin receptor (PAR-1). Bioorg Med Chem Lett.

[B19] Escalante Ascota BA, Amezcua, Gastelum JL, Aldana Alcala I (1994). The vascular effects of thrombin on canine and human arteries and their independence from the metabolism of arachadonic acid. Arch Inst Cardiol Mex.

[B20] Storck J, Zimmermann ER (1996). Regulation of the thrombin receptor response in human endothelial cells. Thromb Res.

[B21] Hamilton JR, Nguyen PB, Cocks TM (1998). Atypical Protease-activated receptor mediates endothelium-dependent relaxation of human coronary arteries. Circ Res.

[B22] Soifer S, Peters K, O'Keefe J, Coughlin S (1994). Disparate temporal expression of the prothrombin and thrombin receptor genes during mouse development. Am J Pathol.

[B23] Bernatowicz MS, Klimas CE, Hartl KS, Peluso Mallagretto NJ, Seiler SM (1996). Development of Potent Thrombin Receptor Antagonist Peptides. J Med Chem.

[B24] O'Sullivan CJ, O'Loughlin AJ, Friel AM, Elliot JT, Morrison JJ (2003). PAR function in human pregnant myometrium: effects of thrombin and specific PAR-1 agonist and antagonist [abstract]. J Soc Gynecol Invest.

